# Early Postoperative Recovery after Modified Ultra-Minimally Invasive Sonography-Guided Thread Carpal Tunnel Release

**DOI:** 10.3390/jpm13040610

**Published:** 2023-03-31

**Authors:** Konrad Mende, Saskia J. M. Kamphuis, Valentin Schmid, Dirk J. Schaefer, Alexandre Kaempfen, Andreas Gohritz

**Affiliations:** Clinic of Plastic, Reconstructive, Aesthetic and Hand Surgery, University Hospital Basel, Spitalstrasse 21, 4031 Basel, Switzerland

**Keywords:** carpal tunnel syndrome, ultrasound, sonography, thread release, minimally invasive, ultra-minimally invasive, percutaneous

## Abstract

Thread carpal tunnel release (TCTR) has been reported to be safe and effective for the treatment of carpal tunnel syndrome. The aim of this study is to evaluate the modified TCTR for safety, efficacy, and postoperative recovery. Seventy-six extremities in 67 patients undergoing TCTR were analyzed pre- and postoperatively using clinical parameters and patient-reported outcome measures. Twenty-nine men and 38 women with a mean age of 59.9 ± 18.9 years underwent TCTR. The mean postoperative time to resume activities of daily living was 5.5 ± 5.5 days, analgesia was completed after 3.7 ± 4.6 days, and return to work was achieved after a mean of 32.6 ± 15.6 days for blue-collar workers and 4.6 ± 4.3 days for white-collar workers. The Boston Carpal Tunnel Questionnaire (BCTQ) and Disability of Arm, Shoulder, and Hand (DASH) scores were comparable with previous studies. Overall, two persistent compressions and one recurrence required open reoperation (3.9%). All three had been operated in the initial phase, and none required reoperation after an additional safety step was introduced. No other complications occurred. TCTR surgery appears to be a safe and reliable technique with almost no wound and scarring and a potentially faster recovery time than open techniques. Although our technical modifications may reduce the risk of incomplete release, TCTR requires both ultrasound and surgical skills and has a considerable learning curve.

## 1. Introduction

Carpal tunnel syndrome (CTS) is caused by compression of the median nerve in the carpal tunnel at the wrist and causes pain, paresthesia, and hypoesthesia in the hand [1]. It accounts for approximately 90% of all entrapment neuropathies and affects up to 10% of the population during their lifetime [2,3,4,5]. 

Early stages of CTS can be treated conservatively with splints, corticosteroid injections, analgesics, or occupational therapy [2,6,7]. If conservative treatment fails, surgery consists of cutting the transverse carpal ligament (TCL) to release the pressure within the carpal tunnel and to reduce compression on the median nerve [8,9]. The classic surgical intervention is open carpal tunnel release (OCTR), but newer methods attempt to reduce surgical morbidity by reducing (mini-open carpal tunnel release, MOCTR) or avoiding (endoscopic carpal tunnel release, ECTR) the often painful scar in the palm [1,6,8,9]. 

In OCTR, the TCL, overlying skin, subcutaneous tissue, superficial palmar fascia, and palmaris brevis muscle of the palm are divided by a 2.5–5 cm incision, allowing direct visualization of all relevant structures [10]. In MOCTR, the incision is reduced to 1–2 cm in the mid palm and the TCL is transected under direct visualization [10,11]. In ECTR, which was introduced in the late 1980s, a 1–1.5 cm transverse skin incision is made proximal to the wrist crease to allow for endoscopic visualization and to create space for a specialized cutting device (hook knife) to transect the TCL while leaving overlying structures intact [12]. The ECTR appears to result in a faster return to daily activities and work, as well as faster incisional healing than OCTR [13]. Disadvantages of the endoscopic method include the narrow view of the surgical field, a steep learning curve, expensive disposable instruments, and its significant set-up time [14]. Technical advances in musculoskeletal ultrasound visualization using high-frequency linear probes, as well as the disadvantages and limitations of OCTR, MOCTR, and ECTR, led to the development of ultra-minimally invasive ultrasound-controlled carpal tunnel release (UMIUCTR), which uses ultrasound for guidance, resulting in lower costs compared to ECTR, as well as safe visualization of all relevant structures and the so-called “safe zone” [15], which is described in detail below. 

One relatively new method, published by Guo et al., is ultrasound-guided thread carpal tunnel release (TCTR) [16]. This procedure uses a special self-developed cutting thread that is not commercially available and not approved for medical use in the European Union (EU). The thread is inserted percutaneously and looped around the TCL under continuous ultrasound visualization, allowing for the “scarless” transection of the TCL without the need for skin closure [14], suture removal, or dressings for longer than a few days. TCTR has been reported to result in a faster return to daily activities, faster return to work, and faster discontinuation of pain medication than ECTR [16]. Until recently, the results of TCTR have been reported exclusively by its developers or collaborators, so there is a lack of independent evaluation [17]. In 2021, Burnham and colleagues, a group of Canadian hand surgeons, reported their outcomes in a case series using a similar technique but a different thread from an industrial supplier [18].

The aim of our study is to evaluate the results of TCTR using the surgical method described by Guo et al. in terms of efficacy, safety, and postoperative recovery using objective and subjective outcome measures including patient-reported outcome measures (PROMs) in a Swiss university hospital setting [16]. As a necessary modification, we used a cutting thread that is both commercially available and approved for medical use in the EU, with a focus on the early postoperative period to allow comparison with other techniques. 

## 2. Materials and Methods

### 2.1. General Introduction

In this retrospective, uncontrolled, unblinded study, the data of all patients with CTS who underwent treatment with TCTR in the Clinic for Plastic, Reconstructive, and Hand Surgery at the University Hospital Basel by senior surgeons (surgical level of expertise III [19]) between 2019 and 2022 were analyzed by clinical chart review. 

Informed consent was obtained from all individuals of this study. Ethical approval was obtained from the local Ethics Committee of Northwest and Central Switzerland (EKNZ). Only patients with a complete six-week follow-up after surgery were included. The diagnosis of CTS was based on standard clinical criteria, including medical history, physical examination, and sonographic and electrophysiological studies. Preoperative and postoperative data were collected at one week, three weeks, six weeks, and six months; a follow-up of at least six weeks was required for inclusion. Complications were graded and recorded as major complications such as neurovascular or musculoskeletal damage, or minor complications such as infection. Revision cases were reviewed separately [20]. 

Data collected included thenar muscle atrophy, two-point sensory discrimination, abductor pollicis brevis muscle strength (Medical Research Council (MRC) grading 0–5), grip and pinch strength (in kilograms), pain intensity levels (Visual Analogue Scale (VAS) 0 = minimum to 10 = maximum), satisfaction (VAS, 0–10), and ultrasound measurement at the cross-sectional area (cm^2^) of the median nerve at distal forearm level and at proximal wrist crease level. Grip strength was defined as the maximal force in full fist closure and pinch strength was the maximal force possible for thumb to index pinch. Time to return to daily activities, return to work, and discontinuation of pain medication were recorded. 

The Boston Carpal Tunnel Questionnaire (BCTQ) and the Disability of Arm, Shoulder, and Hand (DASH) questionnaire were used [21,22] as patient-reported outcomes. Scores from the BCTQ and DASH were calculated as described during the validation process [22,23,24]. For the BCTQ, both the symptom and functional assessment sections were included to calculate the overall score; for the DASH, the sections that fulfilled completeness requirements were included. Procedure time was measured as the time from the first injection of local anesthetic to the end of the postoperative dressing.

### 2.2. Surgical Method

#### 2.2.1. Surgical Equipment

The equipment used for the technique included ultrasound (flexFocus 500, B K medical, Burlington, MA, USA) with an 18 MHz linear probe, 25 G and 23 G cannulas (AGANI ™ Needle, Zhejiang Kindly Medical Devices Co., Zhejiang Province, China), a 27 G 1.5-inch cannula (Sterican^®^, B. Braun Medical AG, Sempach, Switzerland), 18 G 3.5-inch epidural cannulas (Perifix^®^, B. Braun Medical AG, Sempach, Switzerland), 16 G 1.2-inch cannulas (Appli-Set, Applimed S.A., Chatel-Saint-Denis, Switzerland), a cutting thread (FiberStick™, Arthrex GmbH, Muenchen, Germany), and 1% rapidocaine with epinephrine (Sintetica S.A.^©^, Mendrisio, Switzerland) with 10% of sodium bicarbonate 8.4% added (Bichsel^®^, Unterseen, Switzerland).

#### 2.2.2. Surgical Procedure

**Figure 1:**
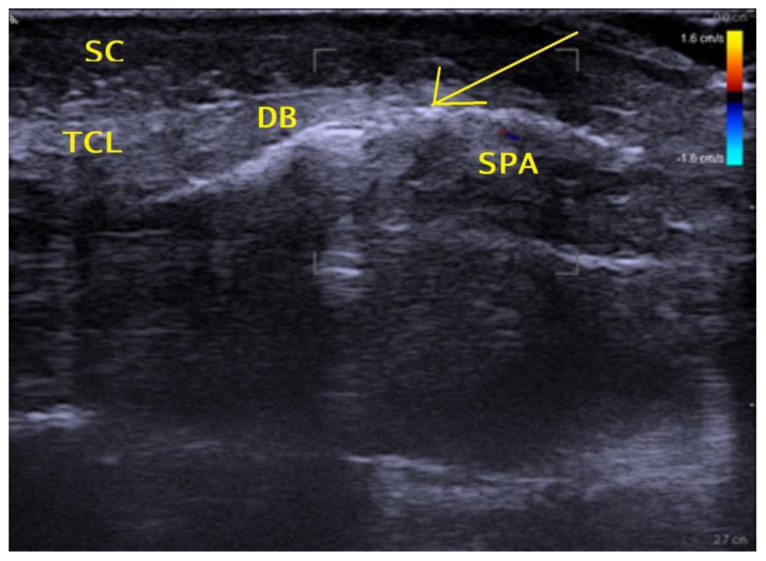
Sagittal distal palmar sonographic view. Arrow = distal needle insertion site; SC = subcutaneous tissue; TCL = transverse carpal ligament; DB = duck’s beak; SPA = superficial palmar arterial arch.

The procedures were performed under local anesthesia without a tourniquet, in an outpatient operating room, by a board-certified hand surgeon, supported by a medical assistant. Patients were conscious and able to cooperate with the surgeon throughout the procedure. Preoperative ultrasound was used to identify the relevant palmar structures of the hand, wrist, and distal forearm, including the course of the median nerve, the common digital nerves of the third and fourth web spaces, the motor branch of the median nerve, a median-ulnar communicating or ‘Berrettini’s’ branch if present, the flexor tendons, the ulnar nerve and artery and the superficial palmar arterial arch (SPA), the proximal and distal margins of the TCL (the latter is also known as the duck’s beak (DB) due to its shape [25]) and the bony landmarks of the pisiform bone, the tubercle of the scaphoid, and the hook of the hamate. The “safe zone” or “safe line”, which represents the area between the ulnar-most limit of the median nerve and the radial limit of the ulnar artery, as described by Nakamichi et al. in 1997 [15], is marked on the skin. The entry point is marked on the skin at the most distal end of the safe zone, immediately distal to the DB and proximal to the SPA. The exit point, which is the proximal limit of dissection and ensures the division of a distal part of the palmar forearm fascia at 2 cm proximal to the wrist crease as a proximal continuation of the safe zone, is equally marked. After sterile preparation and draping, the preoperative markings are again checked and confirmed with the sterile draped ultrasound probe. Using a 27 G 1-inch needle, 1–2 mL of local anesthetic (1% rapidocaine with epinephrine with 10% of sodium bicarbonate 8.4%) was injected subcutaneously at the entry and exit points. A 27 G 2-inch cannula was inserted subcutaneously in the palm at the entry point, advanced proximally using hydrodissection with local anesthetic, and passed over the SPA and through the superficial palmar aponeurosis under the distal edge of the TCL (the DB) into the carpal tunnel space, as shown in Figure 1. 

**Figure 2:**
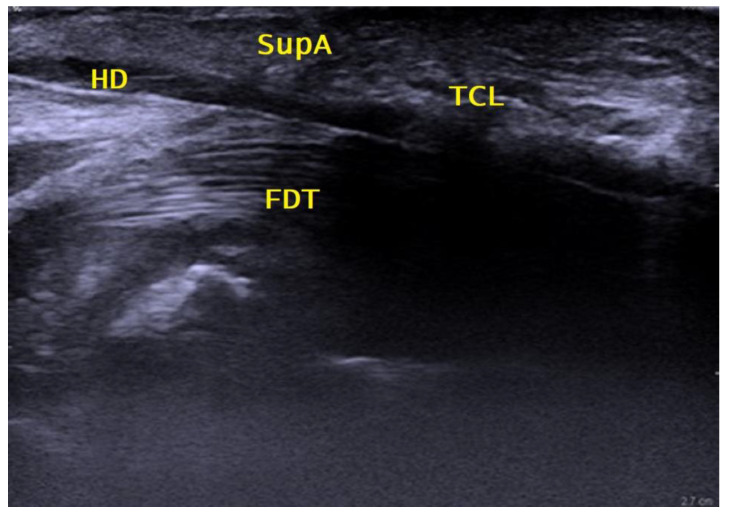
Sagittal proximal palmar view. HD = hydrodissected area; FDT = flexor digitorum profundus and superficialis tendons; TCL = transverse carpal ligament; SupA = superficial palmar aponeurosis.

Another deposit of 1 to 2 mL of local anesthetic was injected to open up the distal carpal tunnel space by hydrodissection. The 27 G cannula was then removed. Under continuous hydrodissection with local anesthetic, a 18 G epidural cannula (slightly bent to match the curved undersurface of the TCL) was then inserted through the same entry point, passed over the SPA and through the palmar aponeurosis under the distal edge of the TCL into the carpal tunnel space. Under continuous real-time ultrasound control, it was then advanced dorsally to the TCL and palmar to the flexor tendons along the safe zone from distal to proximal, exiting at a point 2 cm proximal to the wrist crease, facilitated by slight dorsal extension of the wrist, as shown in Figure 2. The position of the cannula was checked and could be corrected continuously longitudinally and axially over the whole length of the carpal tunnel by sonography to exclude any malpositioning due to movement of the wrist or fingers and the thereby potential positional change of the median nerve in the carpal canal. 

**Figure 3:**
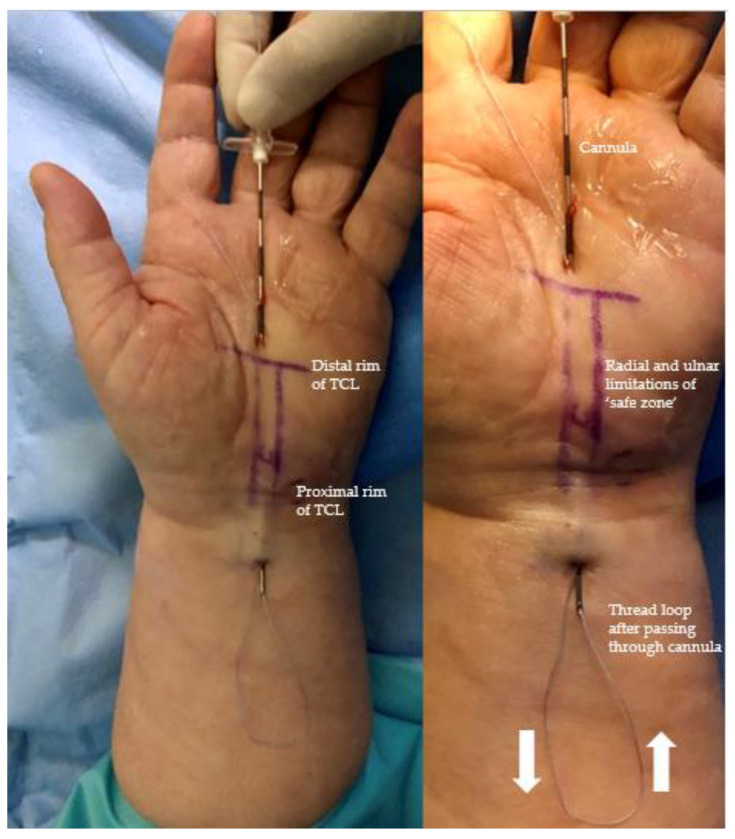
Surgical site after the second pass of the 18 G cannula under the superficial palmar aponeurosis and looping of the thread, including anatomical landmarks and the direction of passage of the thread.

The cutting suture was then inserted and passed through the 18 G epidural cannula from distal to proximal and the cannula was withdrawn, leaving the cutting suture in situ. The suture could then be visualized by sonography on the dorsal surface of the TCL along the safe zone (Figure 3).

Under hydrodissection with local anesthetic and using ultrasound guidance, a straight 18 G epidural cannula was then introduced via the same entry point in the palm, passed along the palmar surface of the TCL (underneath the palmar aponeurosis), exiting through the same exit point in the distal forearm. The cutting suture was then looped back and passed through the cannula from proximal to distal; the cannula was withdrawn, leaving both ends of the cutting thread now emerging in the distal palm. The position of the thread after removal of the cannula can also be checked by sonography; displacement during this process is unlikely or would be detected during the process by sonography. 

The ends were then pulled distally and, under continuous ultrasound visualization, the TCL was transected from proximal to distal, using reciprocal movements of the cutting thread, similar to a Gigli saw. Transmitted motion on adjacent tissue can be seen. If the median nerve or tendons were moved, the thread could be replaced if necessary.

After the procedure, a bulky dressing is applied for the first 24 h and then the patient manages the wound with simple adhesive dressings for another 2–3 days until the puncture wounds are dry. Patients are instructed to commence active mobilization of the hand, including all fingers, the thumb, and the wrist, immediately after surgery. Patients are also instructed that the only limiting factor is pain after surgery, and they should start using the hand in daily life as soon as possible. There are no weight limitations or restrictions postoperatively. 

### 2.3. Statistical Analysis

For statistical analysis, IBM SPSS v28 (IBM Company, Chicago, IL, USA) was used to evaluate two-tailed *t*-tests. Normally distributed variables are presented as the mean and standard deviation (SD). The level of significance was set to a *p*-value of ≤0.05.

## 3. Results

### 3.1. Demographic Data

The study included 76 operated extremities in 67 individual patients over a time period of 24 months. Mean follow-up was 13 ± 8.8 weeks. There were 29 male (43%) and 38 female (57%) patients, with a mean age of 59.9 ± 18.9 years. The dominant hand was operated on in 48 cases (63%). In their professional capacity, 12 patients (17.9%) were white-collar workers, 17 patients (25.4%) were blue-collar workers, and 38 patients (56.7%) were retired or unemployed. Six female patients (9.0%) were pregnant at the time of diagnosis and initial treatment. Nine patients were treated on both extremities; four of them were operated bilaterally in the same session at the specific request of the patient. Ten patients were on anticoagulation and five patients were on platelet aggregation inhibitors due to cardiologic diagnoses. None of these medications were interrupted for the intervention. All patients underwent diagnostic electrophysiologic studies; 15 patients (19.7%) showed mild, 25 patients (23.9%) moderate, and 36 patients (47.4%) severe preoperative findings. At baseline, atrophy of thenar eminence was seen in 19 of 76 extremities (25.0%), and clinical thenar muscle weakness with a muscle power grade of less than 5 was found in 20 of 76 extremities (26.3%). Two-point discrimination (2PD) was diminished (more than five millimeters) in 12 of 76 extremities (15.8%).

### 3.2. Ultrasonographic Investigation

Preoperatively, sonographic investigations showed a cross-sectional ratio of 1.85 ± 0.6 cm^2^, which tended to decrease over time. We did not encounter any Berrettini anastomosis among our patients during outpatient clinic evaluations or intraoperatively. 

### 3.3. Operating Time and Complications

**Figure 4:**
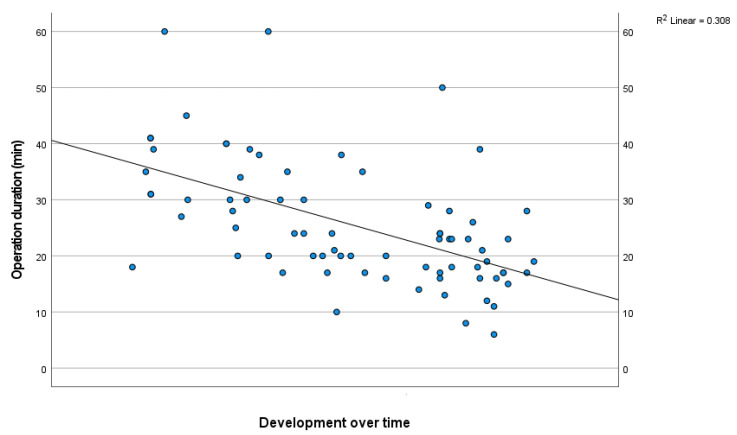
Development of operation duration (minutes) over time.

The mean duration of surgery, including sonographic evaluation and orientation, was 25.5 ± 10.9 min. Over time, the duration of the operation diminished, as shown in Figure 4, which is an indication of the learning curve of this procedure. Three patients underwent reoperation due to recurrent or persisting nerve compression, after 1 week, 7 weeks, and 9 months, respectively. In two cases, the operation was converted intraoperatively to an open release due to unclear anatomy on ultrasound. All patients requiring reoperation were in the first third of the cohort. There were no complications such as infection, nerve damage, or hematomas requiring intervention. 

### 3.4. Grip Strength and PROMs

**Figure 5:**
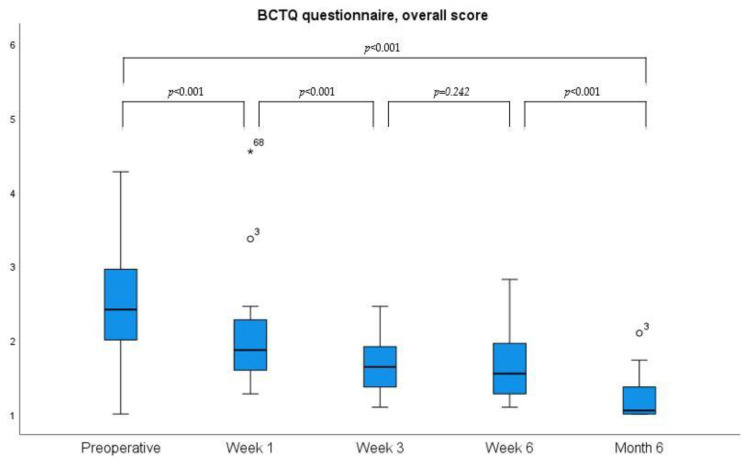
Development of the overall Boston Carpal Tunnel Questionnaire (BCTQ) over time. (* and ° present outliers).

**Figure 6:**
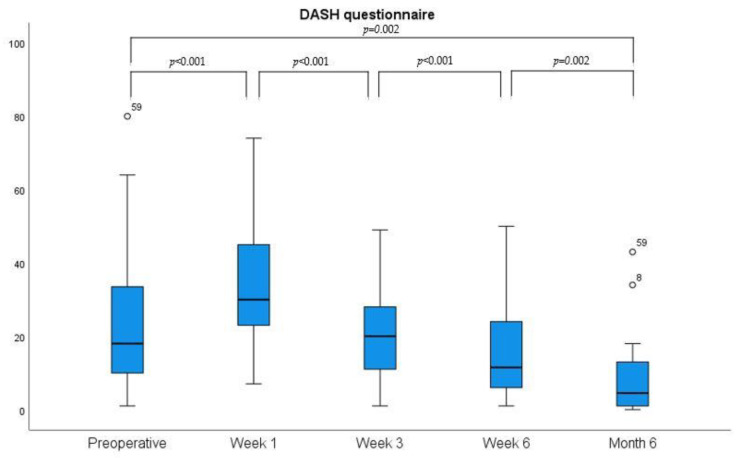
Development the Disabilities of Arm, Shoulder, and Hand (DASH) questionnaire over time. (° present outliers).

Mean preoperative grip strength during hand closure (grasp) was 28 ± 14 kg, 2 ± 7.3 kg one week after surgery, 8 ± 9.4 kg after three weeks, 20 ± 8.7 kg after six weeks, and 24 ± 13.7 kg after six months, respectively. Mean preoperative pinch (thumb to index) strength preoperatively showed a mean of 7.1 ± 2.4 kg, one week after operation 4.5 ± 2.7 kg, at three weeks 6.2 ± 2.0 kg, at six weeks 6.6 ± 2.4 kg, and after six months 7.1 ± 2.7 kg. Overall, there was a continuous improvement in grip strength after an immediate postoperative drop was seen. Both the DASH and the BCTQ questionnaires showed a gradual improvement over time, as shown in Figure 5 and Figure 6. 

### 3.5. Return to Daily Activities, Work, and Satisfaction

Time to complete cessation of analgesia was 3.7 ± 4.6 days. Return to normal activities of daily living was possible after a mean of 5.2 ± 5.5 days. Return to work in a normal capacity was achieved after a mean of 32.6 ± 15.6 days for blue-collar workers (n = 16) and 4.6 ± 4.3 days for white-collar workers (n = 14). Pain intensity (VAS) ranged from 0 to 1 ± 1.7 one week after surgery. Later on, a VAS 0 to 4 ± 1.3 was observed as patients were resuming their full activity. Satisfaction with the treatment was consistently high. One week postoperatively, the range of satisfaction scores was 9 to 10, which was continuous until the end of follow-up. 

## 4. Discussion

The main purpose of this study was to test whether TCTR is a safe and reliable procedure to effectively treat CTS with a slight modification of the original description, including materials freely available on the European market and for safety reasons. 

### 4.1. Safety

Major complications, e.g., median and ulnar nerve injuries (0.10–0.13%), digital nerve injuries (0.39–0.03%), and vessel injuries (0–0.02%), have been reported with open and endoscopic techniques [15,26]. Similar to Guo et al. and Burnham et al., 2021, who used a similar technique with small modifications, as well as Rojo-Manaute et al., 2016, who used a hook knife, no major complications occurred in our study [12,15]. This suggests that minimally invasive procedures are safe and potentially have a lower risk of neurovascular injury compared to open and endoscopic release due to continuous direct visualization. In TCTR, the progress of the TCL division can be safely monitored under ultrasound guidance; with the suture looped around the TCL, it does not injure neurovascular structures and flexor tendons. Similarly, though platelet inhibitors and therapeutic anticoagulation were discontinued in our patients, we did not observe any hematoma or bleeding requiring reintervention. The possibility of continuing platelet aggregation inhibitors was already proven by Brunetti et al. [27]; however, they did not include full therapeutic anticoagulation.

### 4.2. Reoperations for Incomplete Ligament Division

In our cohort, three patients required reoperation for persisting or recurrent nerve compression. At the time of surgery, this cannot be definitively determined, because of artifacts of the local anesthesia and the operation itself. Moreover, the nerve will not return to its original shape immediately after decompression, so a certain hourglass deformation remains at the end of the surgery. In two cases, the complaint did not resolve after initial surgery and persisting nerve compression was verified in sonography or MRI. In the third case, symptoms of median compression at the carpal tunnel recurred after an initial six-month interval without complaints. In this case, electrophysiologic investigation confirmed recurring compression. In the study of Guo et al., no reoperations were described [16]. In open carpal tunnel release, however, reoperation rates are described in up to 4.4%, and in endoscopic release up to 6.5% [28], depending on the experience of the surgeon. Our three patients requiring revision surgery had their original surgery in the first third of our cohort and we believe that the incomplete division of very distal fibers of the TCL in the palm as it approached the SPA was due to the learning curve in these patients. Reoperations for persisting or recurrent CTS symptoms were not required since we adapted our technique by ensuring that the thread was inserted immediately on top of the visualized SPA and immediately diving underneath the very distal duck’s beak. In addition, we began to palpate the divided TCL from the inside at the end of the procedure using a blunt rigid cannula. If any persisting transverse fibers were detected, the procedure was repeated until satisfactory full carpal tunnel release was achieved. We consider this additional step a valuable tool for the verification of complete TCL division, taking minimal extra surgical time and preventing any persisting transverse fibers as a potential cause of persisting nerve compression. We believe that, with more experience, in the future, reoperation rates will be level with the ones published by Lane et al. 2021 at 3.42% [29].

### 4.3. Postoperative Recovery

With an average of 5.2 days, our patients could return to daily life activities five times faster than reported by Rojo-Manaute et al. for open carpal tunnel release and 1.5 times faster than reported for TCL release using a hook knife [30,31]. Guo et al. reported that patients were able to use their hands on the day of the procedure for simple daily activities such as eating, living, or controlling a computer mouse [16]. White-collar workers were able to return to work after 4.6 days and in the case of blue-collar workers after 32.7 days. Blue-collar workers started reintegration by slowly building up their professional activities.

### 4.4. Objective Outcomes and PROMs

We found that grip and pinch strength measurements may not be a valuable outcome parameter to monitor the immediate postoperative follow-up period until week six. While early postoperative grip strength measurements were reduced due to pain caused by direct pressure of the Jamar dynamometer on the operative site (e.g., the divided TCL), pinch strength measurements were not painful, but motor recovery of the thenar muscle seemed not to significantly increase within the first six weeks after CTR. Similarly, 2PD could be used to detect iatrogenic neurovascular injury, but did not show a significant improvement in the early postoperative period in most cases. PROMs, such as the VAS, BCTQ, and the DASH score, may be more useful to monitor postoperative recovery and to detect early symptomatic and functional improvement [26]. High VAS values for satisfaction starting from day one and a very short interval of postoperative pain medication requirement indicate that the procedure is effective and well tolerated. More specifically, we found a significant BCTQ symptom severity improvement one week after TCTR, and Guo et al. reported significant changes as early as day one after the operation. Similarly, our BCTQ scores for function were significantly lower after one week and after three weeks [16]. Burnham et al. had comparable but slightly higher BCTQ scores at week 4 [18]. Therefore, TCTR seems to have the potential for significantly faster postoperative symptom and functional improvements as compared to endoscopic or open procedures when using BCTQ for evaluation. ECTR and OCTR were reported to require 3 weeks for significant symptom relief and 4 to 6 weeks for functional improvement [13,16,18].

### 4.5. Comparison with UMIUCTR Using a Hook Knife

Using the DASH questionnaire, we were able to compare our results to those of a different UMIUCTR method, as reported by the group of Rojo-Manaute et al., who used a retrograde mini hook knife via a small incision. They reported similar DASH scores in their pilot study in 2013 [25]. In their second study in 2016, they reported better DASH scores, although not a clinically important difference (>10.81 point difference), compared to our results [31]. The improvement was in line with our own experience of the learning curve in performance.

### 4.6. Limitations of the Study

The number of treated patients in this study is too low in general and specifically to discuss safety issues that might arise when discussing the detectability of anatomic anomalies such as Berrettini’s anastomosis in the safe zone or an unusually wide ulnar deviation of the motor branch of the median nerve or a median artery. Concerning the communicating or Berrettini’s anastomoses, we did not identify any in our patient cohort. However, in our clinical experience, also in open TCL division, possible Berrettini’s anastomoses are usually more distal and are seldom a risk in the case of TCL division. 

Although this retrospective study shows promising results for this technique also for the European market, future randomized controlled studies comparing the different methods are needed to confirm this finding. 

## 5. Conclusions

In conclusion, TCTR appears to be a safe and reliable technique with the benefits of practically no external wounds and less scarring, and with a potentially faster recovery time, compared to other techniques. The proposed modifications allow for reproducible results and may reduce the risk of persistent nerve compression due to incomplete release. However, TCTR requires both ultrasound and surgical skills and has a considerable learning curve.

Further studies with direct matched comparisons and larger numbers to allow for subgroup analysis are warranted to clarify these advantages.

## Data Availability

The data presented in this study are available on request from the corresponding author. The data are not publicly available due to privacy reasons.
